# Association of dietary insulin index (DII) and dietary insulin load (DIL) with circadian rhythm and quality of sleep among overweight and obese women: a cross-sectional study

**DOI:** 10.1186/s12902-024-01811-1

**Published:** 2024-12-24

**Authors:** Atieh Mirzababaei, Faezeh Abaj, Mina Radmehr, Moloud Ghorbani, Yasaman Aali, Asma Rajabi Harsini, Cain C. T. Clark, Khadijeh Mirzaei

**Affiliations:** 1 Chronic Diseases Research Center, Endocrinology and Metabolism Population Sciences Institute, Tehran University of Medical Sciences, Tehran, Iran; 2 Department of Community Nutrition, School of Nutritional Sciences and Dietetics, Tehran University of Medical Sciences, Tehran, Iran; 3 Department of Nutrition, Dietetics, and Food, Monash University, Melbourne, Australia; 4 Department of Nutrition, Science and Research Branch, Islamic Azad University, Tehran, Iran; 5 Department of Community Nutrition, Faculty of Nutrition and Food Sciences, Tabriz University of Medical Sciences, Tabriz, Iran; 6 Department of Nutrition, Faculty of Medicine, Mashhad University of Medical Science, Mashhad, Iran; 7 Department of Clinical Nutrition, School of Nutritional Sciences and Dietetics, Tehran University of Medical Sciences, Tehran, Iran; 8 Centre for Intelligent Healthcare, Coventry University, Coventry, CV1 5FB UK

**Keywords:** Dietary insulin index, Dietary insulin load, Circadian rhythm, Overweight/Obese women, Quality of sleep

## Abstract

**Background:**

Obesity is a global issue, with over 1.9 billion adults overweight. Disruption of circadian rhythms (CR) leads to obesity and metabolic disorders. Dietary nutrition significantly impacts sleep disorders and disruption in CR, influencing hormones and inflammation, which can contribute to insomnia. The dietary insulin index (DII) and dietary insulin load (DIL) are important factors in determining sleep quality. The current study aims to investigate the association between DII and DIL with CR and sleep quality among with overweight and obesity women.

**Methods:**

A case-control study involved 280 overweight/obese women aged 25–40 from Tehran University Medical Science. They were assessed for dietary intake, physical activity, and sleep using validated questionnaires. The study also assessed body composition, bioelectrical impedance analysis, biochemical components, anthropometric components, and blood pressure. Socio-demographic and lifestyle characteristics, such as age, educational level, physical activity, and smoking habits, were also assessed through questionnaires.

**Result:**

In the crude and adjustment models, high adherence of DII compared with lower adherence increased the odds of poor sleep quality index among participants. This significant association remained even after adjustment for confounding variables (*P* < 0.05), such that the odds of poor sleep quality index was 1.92 times higher.

**Conclusion:**

This study showed high adherence to DII and DIL may cause CR disruption. Furthermore, higher adherence to DII lead to poor sleep quality in women.

## Introduction

The prevalence of obesity has continued to rise globally; according to the World Health Organization (WHO), it is projected that the number of adults living with obesity will increase from 0.81 billion in 2020 to 1.53 billion in 2035 [1]. Industrialization and urbanization have led to a rapid rise in obesity in many Asian countries, particularly in wealthier nations. For instance, China has seen significant increases in both overall and abdominal obesity, especially in urban areas [2]. In South Asia, while undernutrition remains a concern, childhood obesity is also rising, especially among wealthier households [3]. Obesity rates are among the highest globally, especially in the U.S. In 2016, about 40% of American adults were obese, with predictions of further increases by 2030 [4]. In Europe, countries like the U.K. and Greece report high obesity rates, with obesity prevalence reaching up to 20% in some countries [5]. Although the prevalence of obesity is rising in Asia, it is still lower than in the West generally. However, the difference is closing, particularly in Asia’s more affluent and urbanized areas, where a greater emphasis is placed on calorie-dense diets and less physical activity [6].

Obesity is associated with various chronic conditions, including metabolic syndrome, insulin resistance (IR), cardiovascular disease, nonalcoholic fatty liver disease, and certain cancers [7, 8]. Additionally, sleep duration may play a crucial role in regulating body weight and metabolism [9]. Over the past 40 years, daily sleep duration in the U.S. population has decreased by 1 to 2 hours. The proportion of young adults sleeping less than 7 h per night has more than doubled from 1960 to 2001–2002 (from 15.6 to 37.1%) [10–12]. Exposure to artificial light after sunset and before sunrise, disrupting circadian rhythms (CR), has significantly impacted sleep duration. This, in turn, affects overall CR and the ability to be active and eat during the natural night [13]. Numerous studies on sleep and obesity in adults and children conducted in different regions indicate an association between short sleep (generally fewer than 6 h a night) and obesity [14–17]. A meta-analysis showed that for each additional hour of sleep, body mass index (BMI) decreased by 0.35 kg/m^2^ [18]. Obese individuals without obstructive sleep apnea syndrome (OSAS) often experience fragmented and poorer quality sleep compared to non-obese individuals [19]. It has been shown in a previous study that more than 50% of patients with BMI > 42.5 kg/m^2^ suffer from OSAS without reporting any sleep-related disorders [20] and that it can affect the quality of sleep. On the other hand, it is important to note that CR plays an important role in the regulation of sleep-wake cycles, so sleep patterns are affected by these rhythms [21]. The timing of these physiologic rhythms may become altered, leading to changes in the phase relationship of rhythms to each other. When the CR is altered, a person may have difficulty remaining awake, or remaining asleep throughout the desired wake period and falling asleep at a desired time [22]. Moreover, disruption of CR leads to obesity and metabolic disorders [23]. Moreover, a previous study showed [24] that evening chronotype individuals exhibited poorer sleep quality and shorter sleep duration compared with morning chronotype individuals. Also, the authors found that an evening chronotype was associated with a higher BMI, 2 h postprandial blood glucose level, fasting blood glucose level, and IR compared with intermediate and morning chronotype subjects [24]. It is evident that body weight is physiologically regulated, and it is regulated by a set of genes that encode various physiological systems [25]. Nevertheless, it has been proposed that our lifestyles are more responsible for the rapid increase in obesity, as opposed to changes in our genetic milieu over the last few decades. However, recent evidence suggests quality of sleep and CR may also be worthy of attention, for instance, Spiegel et al. [26] and Taheri et al. [27] reported that because of our hectic lifestyles, a lack of sleep can lead to physiological changes in hormonal signals which may promote hunger and then obesity. They showed that insufficient sleep was associated with a reduction in leptin levels, and an increment in ghrelin levels and appetite [26, 27].

Dietary nutrition is believed to significantly impact sleep disorders and disrupt CR [28]. However, a complex relationship exists between sleep and nutritional components. A person’s digestive and metabiotic functions play a significant role in their nutritional factors based on their diet pattern [29]. Furthermore, nutrition exerts a profound influence on hormones and inflammation, both of which can contribute to insomnia directly or indirectly [30–34]. It is widely accepted that the major macronutrient directly linked to postprandial blood sugar elevation is carbohydrates. Consequently, postprandial insulin secretion is primarily influenced by dietary carbohydrates [35]. The Dietary Insulin Index (DII) quantifies insulin responses to isoenergetic components of foods in comparison to the reference food, which is determined based on postprandial insulin secretion [36]. Additionally, aside from considering carbohydrate content, this index analyzes high-protein and high-fat compounds along with their interactions [37]. Dietary Insulin Load (DIL) is another index calculated by multiplying the values of DII for each food by the energy content and frequency of consumption of each food [38]. Excess insulin secretion has been associated with oxidative stress and accelerates beta-cell dysfunction in humans, increasing the risk of type 2 diabetes and obesity [39]. Studies have shown that carbohydrate-rich diets, which elevate postprandial glucose and insulin levels, contribute to the development of IR, an increase in body fat, and undesirable lipid profiles [40–42].

Despite the association of obesity with the quality of sleep and CR evident in previous studies, no study has examined the association between DII and DIL with the quality of sleep and CR. This study explores how dietary factors affect sleep quality and chronic inflammation in overweight and obese women, focusing on the importance of DII in predicting poor sleep quality. Additionally, the study underscores this population’s intricate relationship between dietary habits, sleep quality, and CRs.

## Method

### Study design and population

In this cross-sectional study, a total of 280 overweight/obese women were recruited from health centers affiliated with Tehran University of Medical Sciences in Tehran, Iran, and screened based on the following inclusion/exclusion criteria. This study’s participants were all Iranian women, and no racial or color classification was applied. Additionally, individuals diagnosed with any eating disorder were excluded from the study. The participants were between 25 and 40 years of age with a BMI between 25 and 40 kg/m^2^ [43]. Exclusion criteria included individuals with chronic and debilitating diseases, impaired renal and liver function, thyroid disease, regular use of medication, alcohol or drug abuse, smoking, a history of any type of cancer, pregnancy and/or lactation, menopause, patients with diabetes type 1 and type 2, individuals on calorie restriction diets, or those whose daily energy intake fell outside the range of 800–4200 kcal. Additionally, participants with chronic diseases that could potentially alter dietary intake were excluded. All participants were informed about the study’s aim and procedures and provided written informed consent before participation. Trained investigators administered all questionnaires in this study. The Medical Research Ethics Committee of Tehran University of Medical Sciences approved the study with the following identification: IR.TUMS.MEDICINE.REC.1402.370. Clinical trial number: not applicable.

### Anthropometric measurements assessment

The anthropometric indices measured in this study included weight, height, waist circumference (WC), and hip circumference (HC). Weight was measured to the nearest 100 g using digital scales while the subjects were minimally clothed. Height was measured with a tape measure while the subjects stood erect, without shoes, and with relaxed shoulders. BMI was calculated by dividing the weight (kg) by the square of the height (m). WC and HC were measured with a plastic flexible tape, rounded to the nearest 0.1 centimeters. WC was assessed at the central point of the iliac crest and rib cage, while HC was measured at the largest anterior protrusion. All measurements were conducted by the same professionally trained technician to minimize potential bias.

### Body composition assessment

Body composition was assessed by bioelectrical impedance analysis (BIA), using InBody 770 Body Composition Analyzer. All measurements were conducted in the morning while the participants were fasting. Patients were asked to stand on the Body Composition Analyzer with bare feet and to remove all the accessories containing metal. Body composition was estimated by the prediction equations of manufacturer within the analyzer. All factors that might affect BIA results, including hydration, were considered before measurements [44]. Finally, fat free mass (FFM), body fat mass (BFM), body fat percentage (BF %), and fat mass index (FMI) were measured.

### Laboratory analysis

Fasting blood samples (five mL) (8–10 h of fasting) were collected from the patients to evaluate the serum concentrations of fasting blood glucose (FBG), total cholesterol (TC), triglyceride (TG), high-density lipoprotein cholesterol (HDL-c), and low-density lipoprotein cholesterol (LDL-c). Blood samples were centrifuged and then immediately stored at − 80^°^C until the analyses were performed. All samples were analyzed using a single assay according to the manufacturer’s protocol. The GPO-PAP method was used for measuring TG amounts. TC and HDL-c were calculated using the enzymatic endpoint process and the enzymatic clearance assay [45, 46]. All calculations were made with the usage of a package from Randox Laboratories (Hitachi 902). All measurements were taken at the Endocrinology and Metabolism Research Institute, Bio-nanotechnology Laboratory of Tehran University of Medical Science.

### Assessment of blood pressure

After 15 min of rest, blood pressure (BP) was measured by a trained physician using a standardized sphygmomanometer (Omron, Germany, Europe). Measurements were taken from both arms at 1-minute intervals, and the average of the two readings was calculated to ensure accuracy. This approach helps to account for any variability in blood pressure between the arms, thereby enhancing the reliability of the measurements.

### Other baseline measurements

The socio-demographic and lifestyle characteristics assessed included age, educational level attained, physical activity, and smoking habits by questionnaire. The International Physical Activity Questionnaire (IPAQ) [47] was used to evaluate physical activity. Finally, the level of physical activity recorded by the participants was calculated in terms of the metabolic equivalent of task minutes per week (MET-min/week). To calculate the metabolic equivalent of the task (MET) of each participant, according to the type of activity that was performed, the standard table of MET values was used.

### Dietary assessment

To evaluate the usual dietary intake over the past year, a semi-quantitative food frequency questionnaire (FFQ), containing 147 food items (contains commonly foods consumed by Iranians), was used. The validity and reliability of this questionnaire have already been tested in several studies within the Iranian population [48–50]. Based on predetermined categories, the portion sizes of items consumed in the previous year (daily, weekly, or monthly frequency) were asked. Household measures were used to change portion sizes of the used foods into grams [51]. A predictable average daily food intake parameter (including Carbohydrate, Protein, Total fat, Cholesterol, Saturated fatty acid (SFA), Trans fatty acid (TFA), Monounsaturated fatty acid (MUFA), Polyunsaturated fatty acid (PUFA), Docosahexaenoic acid (DHA), Eicosapentaenoic acid (EPA), Oleic acid, Linoleic acid, Linolenic acid, Total fiber, B1, B2, B3, B5, B6, B9, B12, K, D, E, C, A, Biotin, Sodium, Potassium, Calcium, Iron, Phosphor, Magnesium, Zinc, Chromium, Glucose, Lactose, Maltose and Caffeine.) was computed from the FFQ using NUTRITIONIST IV (N4) software (version 7.0; N-Squared Computing, Salem, OR), modified for Iranian foods.

### Calculation of DII and DIL

The insulin index of each food was extracted for analysis using the methods outlined in the reference [52]. The DII refers to the incremental insulin area under the curve over 2 h in response to the consumption of a 1000-kJ portion of the test food divided by the area under the curve after ingestion of a 1000-kJ portion of the reference food [36]. Test subjects consumed various test foods on separate days, and during the 2 h after consumption, their blood insulin concentrations were measured every 15 min.

To calculate the DIL, the following equation is employed [53]:

insulin load of food= ∑ [insulin index of food × energy content of food(kcal/serving) × frequency of consumption (serving of food/day)].

Moreover, the DII is categorized into two levels based on the cut-off point 38.89. Foods with a DII score of less than 38.89 are classified as low DII adherence, while those with a score of 38.89 or greater are categorized as high DII adherence. Similarly, the DIL is defined using a cut-off point of 9700.31. Foods with a DIL of less than 9700.31 are considered to have low DIL adherence, while those with a DIL of 9700.31 or greater are categorized as high DIL adherence.

### Circadian rhythm and quality of sleep

To assess the CR of participants, we utilized the validated “Morning-Evening Questionnaire” (MEQ). This questionnaire comprises various types of questions, each with specific options and scoring, aiming to evaluate an individual’s mood by inquiring about sleep and wake times, as well as preferred times for engaging in physical and mental activities. The questionnaire presents distinctions in values among the provided alternatives. Scores range from 16 to 86, with higher scores being more prevalent in the morning and lower scores being more common in the evening. Participants were categorized into three groups based on their scores: [1] morning [2, 16–38] normal [39–58], and [3] evening [59–86]. The MEQ has demonstrated validity and reliability in measuring Chronotype in this population [54].

Subjective sleep was evaluated using the Pittsburgh Sleep Quality Index (PSQI), which comprises 19 items scored on a 4-point Likert scale ranging from 0 to 3. The PSQI includes seven subscales: (a) subjective sleep quality, (b) sleep latency, (c) sleep duration, (d) habitual sleep efficiency, (e) sleep disturbances, (f) use of sleeping medication, and (g) daytime dysfunction [55]. To derive the final PSQI score, the seven component scores are summed, resulting in an overall score ranging from 0 to 21, with higher scores indicating poorer sleep quality [56, 57]. The Persian version of the PSQI has been validated and proven reliable in a previous study [55]. The overall Cronbach’s alpha for the PSQI-J global score was 0.77 across all subjects.

### Statistical analysis

The Kolmogorov-Smirnov test was used to determine if the quantitative variables had a normal distribution. Categorical data were represented by percentages and numbers, while continuous variables were represented by mean and Standard Deviations (SDs).

To compare quantitative variables, independent t-tests and Analysis of variance (ANOVA) were used, and chi-square analysis was performed for qualitative variables. Analysis of covariance (ANCOVA) was also used for adjusted analysis, where age, energy intake, and IPAQ were controlled in the adjusted models.

The association between DIL and DII and the risk of poor sleep quality was investigated using binary logistic regression. We also used multinomial logistic regression to investigate the association between DIL and DII with CR.

SPSS software (Version 25, SPSS Inc., Chicago, IL, USA) was used to analyze the data and P-values < 0.05 were considered statistically significant.

## Results

### Study population characteristics

The current study encompassed 280 Iranian women, and the clinical characteristics of the participants are detailed in Table 1. The mean age of the participants was 36.67 ± 9.10 years, with 77.1% being married, 47.5% holding a bachelor’s degree or higher education, and 38.6% being employed. Among the participants, 38.4% reported good sleep quality, while 41.1% experienced poor sleep quality. Additionally, 48.5% of the participants exhibited a normal chronotype, 11.1% identified as morning types, and 22.3% identified as evening types.

**Table 1:** Characteristics of the study population among participants and based on adherence to DII and DIL

Characteristics	DII (*N* = 280)					DIL (*N* = 280)				
	Low (< 38.89) (*N* = 140)	High (≥ 38.89) (*N* = 140)	*P*-value	*P*-value*		Low (< 9700.31) (*N* = 140)	High (≥ 9700.31) (*N* = 140)	*P*-value	*P*-value*	
Age (year)	35.89 ± 8.46	36.85 ± 8.58	0.34	0.35		37.13 ± 8.63	35.63 ± 8.38	0.14	0.14	
PA (min/day)	1151.48 ± 1702.46	1201.06 ± 2310.77	0.84	0.84		1165.37 ± 2011.52	1186 ± 2030.63	0.93	0.33	
Anthropometric indices										
Body weight (kg)	80.53 ± 11.09	79.30 ± 10.84	0.34	0.85		78.98 ± 10.44	80.87 ± 11.43	0.15	0.77	
Height (cm)	161.32 ± 5.49	161.32 ± 6.19	0.99	0.85		160.60 ± 5.84	162.04 ± 5.76	0.03	0.14	
BMI (kg/m^2^)	30.90 ± 3.88	30.56 ± 3.63	0.46	0.86		30.57 ± 3.65	30.88 ± 3.86	0.49	0.27	
Fat mass (kg)	33.85 ± 7.78	33.00 ± 7.58	0.35	0.93		32.91 ± 7.33	33.93 ± 8.02	0.26	0.42	
BF (%)	41.70 ± 5.02	40.88 ± 5.57	0.20	0.93		41.52 ± 5.07	41.06 ± 5.55	0.46	0.27	
FMI (kg/m)	13.09 ± 3.08	12.75 ± 2.91	0.34	0.96		12.88 ± 2.97	12.95 ± 3.03	0.85	0.25	
FFM (kg)	46.57 ± 5.25	46.58 ± 5.56	0.99	0.90		45.82 ± 5.32	47.33 ± 5.39	0.01	0.54	
WC (cm)	99.03 ± 9.70	97.82 ± 9.07	0.28	0.68		97.88 ± 9.27	98.97 ± 9.51	0.33	0.43	
WHR (cm)	0.93 ± 0.05	1.57 ± 7.69	0.32	0.41		1.58 ± 7.72	0.93 ± 0.04	0.65	0.29	
Blood parameters										
FBG (mg/dl)	87.05 ± 9.51	87.51 ± 9.97	0.71	0.74		87.43 ± 9.60	87.12 ± 9.88	0.80	0.94	
TC (mg/dl)	183.85 ± 34.72	184.05 ± 36.76	0.96	0.58		186.60 ± 38.33	181.16 ± 32.60	0.24	0.06	
TG (mg/dl)	124.01 ± 62.76	117.02 ± 58.29	0.67	0.92		120.86 ± 58.27	119.27 ± 62.74	0.15	0.69	
HDL-c (mg/dl)	47.43 ± 9.35	45.99 ± 11.62	0.29	0.49		47.22 ± 11.15	46.17 ± 9.93	0.44	0.81	
LDL-c (mg/dl)	95.90 ± 24.25	93.05 ± 23.51	0.35	0.85		95.19 ± 25.10	93.73 ± 22.60	0.64	0.18	
Blood pressure										
SBP (mmHg)	110.007 ± 13.23	112.10 ± 13.52	0.20	0.57		111.007 ± 13.41	111.09 ± 13.42	0.95	0.97	
DBP (mmHg)	76.58 ± 9.68	78.51 ± 9.45	0.09	0.94		77.49 ± 8.99	77.60 ± 10.19	0.92	0.30	
Qualitative variables										
Marital situation										
Single	29 (45.3)	35 (54.7)	0.39		0.40	28 (43.8)	36 (56.3)	0.25		0.26
Married	111 (51.4)	105 (48.6)				112 (51.9)	104 (48.1)			
Education										
Less diploma	23 (57.5)	17 (42.5)	0.45		0.44	24 (60)	16 (40)	0.33		0.33
Diploma	54 (51.4)	51 (48.6)				53 (50.5)	52 (49.5)			
Bachelor or higher	62 (46.6)	71 (53.4)				62 (46.6)	71 (53.4)			
Job										
Employed	51 (47.2)	57 (52.8)	0.45		0.46	60 (55.6)	48 (44.4)	0.13		0.11
Unemployed	86 (51.8)	80 (48.2)				77 (46.4)	89 (53.6)			
Economic status										
Poor	36 (58.1)	26 (41.9)	0.12		0.13	38 (61.3)	24 (38.7)	0.05		0.05
Moderate	58 (43.6)	75 (56.4)				57 (42.9)	76 (57.1)			
Good	38 (53.5)	33 (46.5)				37 (52.1)	34 (47.9)			

DII: dietary insulin index, DIL: dietary insulin load, PA: physical activity, BMI: body mass index, BF: body fat, FMI: fat mass index, FFM: fat free mass, WC: waist circumference, WHR: weight to hip ratio, FBG: fasting blood glucose, TC: total cholesterol, TG: triglyceride, HDL−c: high−density lipoprotein, LDL−c: low−density lipoprotein, SBP: systolic blood pressure, DBP: diastolic blood pressure

Quantitative variables as means ± SD obtained from the independent t−test

Qualitative variables N (%) obtained from the chi−square analysis

P−value* obtained from ANCOVA test. P−values < 0.05 were considered significant

P−value* for adjustment model, based on age, energy intake and IPAQ

### Study population characteristics according on adherence of DII and DIL

In this study, we used ANCOVA to examine the characteristics of the study population among participants based on adherence to DII and DIL.

ANCOVA allows us to assess the effect of independent variables on a dependent variable while controlling for the influence of confounding variables. In this study, we employed two models: The crude model examines the association between DII and DIL with CR and sleep quality among overweight and obese women without adjusting for confounding variables. The adjusted model refines the analysis by accounting for potential confounding factors, precisely age, energy intake, and IPAQ.

In both the crude and adjusted models, no significant differences were observed in physical activity, anthropometric indices and blood parameters between participants with high adherence to DII and those with low adherence (P-value > 0.05) (see Table 1). In the crude model, there was a higher mean of FFM in subjects with high adherence compared to those with low adherence to DII (P-value = 0.01). However, this significant difference was attenuated after adjusting for confounding variables. In the adjusted model, the mean TC levels were lower in participants with high adherence compared to those with low adherence to DII, although this difference approached but did not reach statistical significance (P-value = 0.06) (see Table 1).

The crude model examines the association between DII and DIL with CR and sleep quality among overweight and obese women without adjusting for confounding variables. This model provides initial insights into the relationships present in the data. The adjusted model refines the analysis by accounting for potential confounding factors, precisely age, energy intake, and physical activity, as assessed by IPAQ. By controlling for these influencing variables, this model aims to evaluate better the associations between DII, DIL, CR, and sleep quality.

### Dietary intakes of population according on adherence of DII and DIL

In Table 2, dietary intake of the study participants based on higher or lower adherence of DII and DIL is presented. There was significant mean difference for total fat, saturated fat, MUFA, PUFA, DHA, EPA, oleic acid, linoleic acid, linolenic acid, Vit E, and Vit A were significantly lower in participants with high adherence of DII versus to those with low adherence. Also, there was significant mean difference for carbohydrates, B1, B5, B9, biotin, iron, magnesium, and chromium, which were significantly higher in greater adherence versus lower adherence of DII (P-value < 0.05).

**Table 2:** Dietary intakes of the study population among participants and based on adherence to DII and DIL

Variables		DII (*N* = 280)			DIL (*N* = 280)		
		Low (< 38.89) (*N* = 140)	High (≥ 38.89) (*N* = 140)	*P*-value*	Low (< 9700.31) (*N* = 140)	High (≥ 9700.31) (*N* = 140)	*P*-value*
Macronutrients							
CHO (gr/ day)		337.24 ± 110.10	400.70 ± 116.89	< 0.001	281.11 ± 62.90	456.24 ± 91.56	0.001
Pro (gr/day)		87.39 ± 32.33	89.56 ± 26.48	0.34	69.93 ± 17.93	107.12 ± 27.20	0.41
Tfat (gr/day)		99.48 ± 34.76	87.12 ± 28.61	< 0.001	77.62 ± 26.99	109.37 ± 29.60	< 0.001
Subgroup types of fat							
Chol (gr/day)		260.49 ± 127.72	244.43 ± 80.61	0.01	205.64 ± 67.94	300.01 ± 118.65	0.74
SFA (gr/day)		27.96 ± 11.75	27.35 ± 10.49	0.04	22.01 ± 7.83	33.36 ± 11.09	0.47
TFA (gr/day)		0.001 ± 0.003	0.0008 ± 0.001	0.18	0.001 ± 0.003	0.001 ± 0.002	0.19
MUFA (gr/day)		33.53 ± 12.89	28.70 ± 9.12	< 0.001	26.86 ± 11.20	35.51 ± 9.97	0.001
PUFA (gr/day)		22.31 ± 9.71	17.50 ± 6.59	< 0.001	17.88 ± 9.06	22.05 ± 7.71	< 0.001
DHA (gr/day)		0.12 ± 0.13	0.09 ± 0.10	0.03	0.09 ± 0.09	0.13 ± 0.14	0.64
EPA (gr/day)		0.03 ± 0.04	0.02 ± 0.03	0.04	0.02 ± 0.03	0.03 ± 0.04	0.54
Oleic acid (gr/day)		30.16 ± 12.43	25.68 ± 8.43	< 0.001	24.29 ± 11.009	31.67 ± 9.43	0.002
Linoleic acid (gr/day)		19.30 ± 9.50	15.10 ± 6.10	< 0.001	15.55 ± 8.74	18.96 ± 7.44	0.001
Linolenic acid (gr/day)		1.32 ± 0.64	1.07 ± 0.54	< 0.001	1.04 ± 0.62	1.35 ± 0.54	0.01
Total fiber (gr/day)		43.91 ± 19.59	46.23 ± 18.51	0.74	35.95 ± 15.38	54.21 ± 18.06	0.66
Micronutrients							
Vitamins							
B1 (mg/day)		1.88 ± 0.60	2.26 ± 0.66	< 0.001	1.59 ± 0.38	2.54 ± 0.53	0.001
B2 (mg/day)		2.10 ± 0.81	2.25 ± 0.77	0.48	1.70 ± 0.52	2.65 ± 0.73	0.29
B3 (mg/day)		24.59 ± 10.31	26.03 ± 8.53	0.85	19.79 ± 5.17	30.84 ± 9.64	0.63
B5 (mg/day)		6.01 ± 2.08	6.95 ± 2.79	0.005	5.07 ± 1.37	7.89 ± 2.57	0.17
B6 (mg/day)		2.06 ± 0.76	2.26 ± 0.67	0.14	1.71 ± 0.47	2.62 ± 0.65	0.14
B9 (mg/day)		571.11 ± 170.07	633.27 ± 183.46	0.01	488.79 ± 120.40	715.24 ± 155.30	0.19
B12 (mg/day)		4.57 ± 3.04	4.20 ± 1.81	0.05	3.46 ± 1.58	5.32 ± 2.91	0.98
K (mg/day)		220.24 ± 253.80	211.14 ± 138.97	0.50	197.99 ± 230.16	233.73 ± 176.07	0.38
D (µ/day)		2.12 ± 1.77	1.88 ± 1.62	0.11	1.60 ± 1.27	2.40 ± 1.96	0.78
E (mg/day)		19.76 ± 11.20	15.11 ± 6.02	< 0.001	16.46 ± 9.96	18.52 ± 8.54	0.001
C (mg/day)		171.92 ± 98.96	205.38 ± 114.69	0.06	146.29 ± 88.82	230.67 ± 109.45	0.36
A (mg/day)		815.57 ± 493.96	751.66 ± 333.35	0.02	663.83 ± 378.56	905.72 ± 432.82	0.28
Biotin (mg/day)		35.53 ± 13.89	41.56 ± 20.26	0.02	30.25 ± 11.10	46.78 ± 18.90	0.11
Minerals							
Sodium (mg/day)		4221.48 ± 1470.53	4234.40 ± 1436.89	0.27	3523.62 ± 1044.50	4937.86 ± 1460.90	0.97
Potassium (mg/day)		4143.57 ± 1541.33	4456.58 ± 1560.19	0.52	3416.77 ± 1190.32	5184.22 ± 1366.78	0.47
Calcium (mg/day)		1111.52 ± 440.04	1202.83 ± 412.82	0.37	913.99 ± 322.09	1400.49 ± 381.91	0.18
Iron (mg/day)		17.44 ± 5.75	19.68 ± 6.01	0.001	14.35 ± 3.51	22.75 ± 4.88	0.02
Phosphor (mg/day)		1552.64 ± 538.31	1704.33 ± 511.66	0.06	1272.50 ± 343.41	1984.27 ± 436.72	0.01
Magnesium (mg/day)		428.51 ± 142.32	486.65 ± 148.77	0.002	361.10 ± 112.47	553.65 ± 112.74	0.05
Zinc (mg/day)		12.22 ± 4.20	13.41 ± 4.20	0.05	9.93 ± 2.75	15.70 ± 3.43	0.05
Chromium (mg/day)		0.08 ± 0.05	0.13 ± 0.09	< 0.001	0.07 ± 0.05	0.14 ± 0.09	0.002
Other							
Glucose (gr/day)	19.12 ± 12.68		22.34 ± 10.55	0.17	15.62 ± 8.009	25.80 ± 12.75	0.96
Lactose (gr/day)	14.69 ± 10.94		15.02 ± 10.37	0.79	11.64 ± 8.86	18.09 ± 11.32	0.91
Maltose (gr/day)	1.73 ± 2.05		1.82 ± 0.93	0.72	1.27 ± 0.61	2.28 ± 2.07	0.63
Caffeine		135.77 ± 100.25	161.09 ± 196.41	0.35	132.58 ± 193.67	163.88 ± 101.18	0.51

DII: dietary insulin index, DIL: dietary insulin load, CHO: Carbohydrates, Pro: Protein, Tfat: total fat, SFA: Saturated fatty acid, TFA: Trans fatty acid, Chol: cholesterol, MUFA: monounsaturated fatty acid, PUFA: polyunsaturated fatty acid, EPA: eicosapentaenoic acid, DHA: docosahexaenoic acid

The use of ANCOVA test

All data are presented as mean ± SD

P−value * is obtained by adjusting the variables of age, energy intake and IPAQ, and < 0.05 were considered significant

There was a significant mean difference for carbohydrates, total fat, MUFA, PUFA, oleic acid, linoleic acid, linolenic acid, B1, Vit E, iron, phosphor, and chromium, which were significantly higher in participants with high adherence versus those with low adherence of DIL (P-value < 0.05).

### Characteristics of study population according to sleep quality index and circadian rhythm

In the Table 3, there was a significant difference between sleep quality index and marital situation in both crude and adjusted models (P-value < 0.05).

**Table 3:** Characteristics of the study population among participants and based on sleep quality index and circadian rhythm

Characteristics	Sleep quality index (*N* = 321)				Circadian rhythm (*N* = 330)				
	< 5 Good (*N* = 155)	≥ 5 Poor (*N* = 166)	*P*-value	*P*-value*	Normal (*N* = 196)	Morning (*N* = 45)	Evening (*N* = 90)	*P*-value	*P*-value*
Age (year)	36.35 ± 8.60	36.84 ± 10.16	0.64	0.65	36.29 ± 9.81	34.11 ± 8.10	39.02 ± 8.51	0.009	0.007
PA (min/day)	961.64 ± 1014.94	1212.95 ± 1978.24	0.25	0.3	1087.48 ± 1665.42	890.51 ± 1090.18	1488.15 ± 2511.28	0.28	0.19
Anthropometric indices and body composition									
Body weight (kg)	80.93 ± 12.64	80.87 ± 11.91	0.96	0.98	80.62 ± 11.97	84.55 ± 14.72	79.25 ± 10.71	0.05	0.17
Height (cm)	160.86 ± 5.99	161.14 ± 5.86	0.67	0.99	160.56 ± 5.76	162.82 ± 6.03	160.70 ± 5.91	0.06	0.89
BMI (kg/m^2^)	31.20 ± 4.31	31.18 ± 4.23	0.96	0.87	31.31 ± 4.30	31.81 ± 4.49	30.70 ± 3.85	0.31	0.08
Fat mass (kg)	34.35 ± 8.59	34.93 ± 8.83	0.54	0.78	34.78 ± 8.48	36.88 ± 10.82	33.21 ± 7.56	0.06	0.11
BF (%)	42.05 ± 5.28	42.70 ± 5.41	0.28	0.66	42.71 ± 5.33	42.84 ± 5.63	41.49 ± 5.03	0.16	0.22
FMI (kg/m)	13.30 ± 3.30	13.54 ± 3.43	0.52	0.74	13.58 ± 3.35	13.89 ± 3.76	12.88 ± 3.02	0.16	0.18
FFM (kg)	46.49 ± 6.02	46.00 ± 5.40	0.43	0.71	45.87 ± 5.68	47.81 ± 5.78	46.18 ± 5.43	0.11	0.46
WC (cm)	98.98 ± 10.50	99.51 ± 9.72	0.63	0.57	99.33 ± 9.81	102.22 ± 11.48	97.81 ± 9.54	0.05	0.09
WHR (cm)	0.93 ± 0.05	1.48 ± 7.06	0.33	0.21	0.93 ± 0.05	0.94 ± 0.05	1.93 ± 9.60	0.27	0.22
Blood parameters									
FBG (mg/dl)	87.46 ± 9.45	86.79 ± 9.68	0.63	0.70	87.32 ± 10.10	87.47 ± 8.55	86.79 ± 8.57	0.93	0.68
TC (mg/dl)	184.66 ± 30.49	176.97 ± 33.46	0.10	0.41	178.21 ± 31.64	188.69 ± 47.79	186.65 ± 32.61	0.19	0.10
TG (mg/dl)	108.57 ± 54.05	124.80 ± 61.5	0.04	0.06	116.24 ± 58.79	114.76 ± 55.02	120.25 ± 60.17	0.89	0.57
HDL-c (mg/dl)	47.85 ± 9.80	46.20 ± 9.27	0.24	0.08	46.79 ± 8.63	45.52 ± 12.16	48.15 ± 10.87	0.50	0.63
LDL-c (mg/dl)	100.97 ± 21.56	95.33 ± 22.67	0.08	0.49	95.99 ± 22.33	97.47 ± 28.28	102.36 ± 21.30	0.22	0.84
Blood pressure									
SBP (mmHg)	111.32 ± 12.97	110.03 ± 16.62	0.51	0.57	110.39 ± 15.23	109.53 ± 14.31	112.89 ± 14.52	0.46	0.59
DBP (mmHg)	78.05 ± 8.45	76.24 ± 12.00	0.20	0.29	77.20 ± 10.79	74.57 ± 7.72	78.47 ± 9.36	0.24	0.17
Qualitative variables									
Marital situation									
Single	33 (34.7)	62 (63.5)	0.002	0.002	36 (70.6)	6 (11.8)	9 (17.6)	0.06	0.06
Married	122 (54)	104 (46)			92 (53.8)	21 (12.3)	58 (33.9)		
Education									
Less diploma	25 (58.1)	18 (41.9)	0.38	0.40	19 (55.9)	1 (2.9)	14 (41.2)	0.21	0.22
Diploma	57 (46.7)	65 (53.3)			50 (59.5)	9 (10.7)	25 (29.8)		
Bachelor or higher	73 (46.8)	83 (53.2)			59 (56.7)	17 (16.3)	28 (26.9)		
Job									
Employed	56 (42.7)	75 (57.3)	0.10	0.12	51 (60.7)	6 (7.1)	27 (32.1)	0.18	0.2
Unemployed	97 (51.9)	90 (48.1)			74 (54.8)	21 (15.6)	40 (29.6)		
Economic status									
Poor	28 (58.3)	20 (41.7)	0.29	0.29	30 (61.2)	2 (4.1)	17 (34.7)	0.19	0.17
Moderate	52 (51)	50 (49)			60 (54.1)	18 (16.2)	33 (29.7)		
Good	35 (63.6)	20 (36.4)			37 (64.9)	6 (10.5)	14 (24.6)		

DII: dietary insulin index, DIL: dietary insulin load, PA: physical activity, BMI: body mass index, BF: body fat, FMI: fat mass index, FFM: fat free mass, WC: waist circumference, WHR: weight to hip ratio, FBG: fasting blood glucose, TC: total cholesterol, TG: triglyceride, HDL−c: high−density lipoprotein, LDL−c: low−density lipoprotein, SBP: systolic blood pressure, DBP: diastolic blood pressure

Quantitative variables as means ± SD obtained from the independent t−test

Qualitative variables N (%) obtained from the chi−square analysis

P−value* obtained from ANCOVA test

P−value * is obtained by adjusting the variables of age, energy intake and IPAQ, and < 0.05 were considered significant

In the crude model, we found a significant difference between sleep quality index and TG level (P-value = 0.04), but in the adjusted model, we observed a marginal significant association (P-value = 0.06). Such that there was an association between poor sleep quality index and higher mean of TG levels.

In the crude model, there was a marginal significant difference between CR and body weight (P-value = 0.05), WC (P-value = 0.05), and height and marital situation (P-value = 0.06). In the adjusted model, there was a significant difference between CR and age (P-value = 0.007), and also a marginal significant difference for marital situation (P-value = 0.06).

### The association between adherence of DII and DIL with sleep quality index

Binary logistic regression was used, in both crude and adjusted models (for confounder potential variables such as age, energy intake and IPAQ), to assess the association between DII and DIL with sleep quality index in women (Table 4).

**Table 4:** The association between higher adherence of DII and DIL with sleep quality index and circadian rhythm status

Variables	Higher of DII (*N* = 280)						Higher of DIL (*N* = 280)					
	Crude model			Adjust model			Crude model			Adjust model		
	OR	0.95% CI	*P*-value	OR	0.95% CI	*P*-value	OR	0.95% CI	*P*-value	OR	0.95% CI	*P*-value
Sleep quality index												
**< 5 Good**	Reference group											
**≥ 5 Poor**	**1.83**	**1.05**,** 3.18**	0.03	**1.92**	**1.04**,** 3.55**	0.03	**1.11**	**0.64**,** 1.91**	**0.69**	**1.41**	**0.58**,** 3.45**	**0.44**
Circadian rhythm												
**Normal**	Reference group											
**Morning**	**0.92**	**0.40**,** 2.11**	**0.84**	**0.69**	**0.27**,** 1.73**	**0.42**	**2.09**	**0.85**,** 5.13**	**0.10**	**1.02**	**0.25**,** 4.05**	**0.97**
**Evening**	**1.61**	**0.33**,** 1.11**	**0.10**	**1.51**	**0.26**,**1.009**	0.05	**1.52**	**0.28**,** 0.96**	0.03	**1.31**	**0.11**,** 0.85**	0.02

DII: dietary insulin index, DIL: dietary insulin load

All data are presented as OR and 95% Cl by binary logistic regression

Adjustment model: based on age, energy intake and IPAQ

Low adherence of DII and DIL, good sleep quality, and normal circadian rhythm group were considered as reference group

P−values ≤ 0.05 were considered significant

In the crude model, high adherence of DII (OR: 1.83, 95%CI: 1.05,3.18; P-value = 0.03) compared with lower adherence, increased the odds of poor sleep quality index among participants. This significant association remained even after adjustment for confounding variables (OR: 1.92, 95%CI: 1.04,3.55; P-value = 0.03), such that the odds of poor sleep quality index was 1.92 times higher.

No significant difference between higher adherence of DIL versus lower adherence and the odds of poor sleep quality index was seen in the crude or adjusted models (P-value > 0.05).

### The association between adherence of DII and DIL with circadian rhythm status

There were no observed significant associations between higher adherence versus lower adherence of DII with CR for morning type, as compared to normal chronotype before and after adjusting for confounders (P-value > 0.05).

In the adjusted model, high adherence of DII (OR: 1.51, 95%CI: 0.26, 1.009; P-value = 0.05), compared with lower adherence, increased the odds of CR for evening type compared to normal type participants.

There were no significant associations between higher adherence versus lower adherence of DIL with CR in morning type vs. normal type, in crude and adjusted models (P-value > 0.05).

In the crude model, high adherence of DIL (OR: 1.52, 95%CI: 0.28,0.96; P-value = 0.03) compare with lower adherence, increased the odds of evening type compare to normal type among participants. This significant relation remained even after adjustment for confounding variables (OR: 1.31, 95%CI: 0.11, 0.85; P-value = 0.02), so that the odds of evening type compare to normal type was 1.31 times higher (Table 4).

## Discussion

Obesity and overweight among Iranian women are an essential and increasing public health issue. According to studies, the rate of overweight and obese Iranian women is very high [58]. In 2021, a study reported that the prevalence of obesity in Iranian women was 24.96%, and overweight and obesity included a total of 63.02% of the female population [59]. This increases the risk of related chronic diseases such as diabetes, heart disease, high blood pressure, and cancer [60, 61]. Hormones play an essential role in the occurrence of obesity in women, and their fluctuations throughout women’s lives affect obesity and the way body fat is distributed. A decrease in estrogen levels, especially after menopause, causes an increase in abdominal fat and a decrease in muscle mass. These changes lead to an increased risk of heart disease and diabetes [62, 63].

The extant literature suggests an association between obesity and CR and sleep quality. However, this is confounded by the dearth of data considering the impact of DII and DIL. Therefore, we conducted the present study to examine the association between DII and DIL with CR and sleep quality among overweight and obese women.

Accordingly, our results demonstrated that high adherence to DII, compared with lower adherence, increased the odds of evening chronotype versus normal. Also, subjects with higher adherence to DII had increased odds of poor sleep quality index compared with lower adherence. Despite the decreasing OR, there were no significant associations between higher adherence compared to lower adherence of DII with CR in morning type vs. normal type; this may be due to the influence of other factors beyond food intake on this association. High adherence to DIL, compared with lower adherence, increased the odds of evening type compared to normal type among participants. Despite an increasing OR, no significant difference was seen between higher adherence to DIL versus lower adherence and the odds of poor sleep quality index. Moreover, no significant statistical association was seen between higher adherence to DIL versus lower adherence to CR for morning type compared to normal type. These findings might be explained by the process of insulin discharge, which is regulated by various factors, including hormones, other nutrients, and neuronal inputs [64].

Although studies in this area were limited, findings from a study on healthy adults demonstrated that consuming a high-glycemic Index (GI) meal four hours before bedtime yielded a significant decrease in sleep onset latency compared to subjects consuming a low-GI meal [65]. A cohort study with a 3-year follow-up showed that high-GI diets could be a risk factor for insomnia in postmenopausal women [66]. In a cross-over study on male athletes, total sleep time and efficiency were higher in the high-GI compared to the low-GI group [67]. In another cross-over study, meal consumption with a high GI did not effect on quantitative and qualitative sleep variables in male athletes [68].

In a cross-sectional study, which was carried out in 2021, subjects in the highest quartile of dietary Glycemic Load (GL) had higher odds of long sleep periods compared to individuals in the lowest quartile [69]. Results from a recent study showed that higher adherence to diets with greater GL could increase the odds of insomnia in participants [70]. In another cross-sectional study conducted on overweight and obese women, higher adherence to a low carbohydrate diet lowered the odds of circadian rhythm disruption in morning-type individuals [71]. Results from a multicenter survey entitled “Three-generation Study of Women on Diets and Health” in Japan demonstrated that poor sleep quality was related to high carbohydrate intake in middle-aged female workers [72]. Whilst in the “Bogalusa Heart Study”,3 years of follow-up data suggested that evening chronotype was independently related to obesity [73]. In a cross-sectional study performed in 2022, high adherence to the Mediterranean diet among university students was positively associated with morning chronotype and better sleep quality [74]. Results from the “Opera Prevention Project” among Italian adults showed that the evening chronotype was associated with obesity and low adherence to the Mediterranean dietary pattern [75]. Also, it has been demonstrated that adherence to a Mediterranean diet has a suitable effect on sleep quality [76]. In a study by Rawat et al., participants of the evening chronotype had a significant positive association of IR with poor sleep quality compared to subjects of intermediate and morning chronotypes [24]. Additionally, the “National FINRISK 2007 Study” showed that the evening-type individuals had lower adherence to a healthy Nordic diet [77].

It is important to note that the studies mentioned were conducted in diverse populations, featuring distinct sample sizes and study designs. Therefore, it is crucial to approach the results with caution and carefully consider potential heterogeneity between studies.

Several studies were performed to determine the association of dietary nutrition with sleep disorders. About macronutrients, high carbohydrate and fat in diet, high-saturated fat and low fiber were associated with lighter sleep and poorer sleep quality [78, 79]. Also, Carbohydrate and protein deficits were related to shorter sleep duration, and micronutrient consumption influenced sleep patterns. For example, deficiencies in iron, zinc, magnesium, selenium, vitamin B1, folate, and phosphorus were associated with shorter sleep duration [80]. Lack of calcium, selenium, and alpha-carotene were related to difficulty falling asleep, while low intakes of vitamin D and lycopene were associated with sleep preservation [81]. The disruption of the sleep-wake cycle can be the primary cause of several diseases like impaired glucose tolerance, diabetes, obesity, premature mortality, and psychiatric disorders, such as depression, anxiety, loss of concentration, cancer progression and tiredness [82, 83].

The relationship between IR, obesity, and CR is regulated through complex pathways, especially in obese individuals. Scientific studies show that the CR and the sleep-wake cycle play a crucial role in regulating energy metabolism, insulin secretion, and, as a result, sleep quality. Studies have shown that disruption of the CR can lead to IR and obesity. In this context, the lack of coordination of the insulin rhythm with the day and night activities of the body can lead to a decrease in insulin sensitivity. In mice with disrupted CR, IR increased, and metabolic regulation was impaired [84]. Insufficient sleep or waking up at inappropriate times can reduce insulin secretion and disrupt glucose metabolism, leading to prediabetes in obese individuals. A study showed that sleep deprivation, along with CR disruption, can lead to insufficient insulin secretion and increased blood glucose levels, which significantly increases the risk of obesity and diabetes [85]. In obese people, disruption of the CR causes hormonal changes that lead to an increase in the secretion of hormones such as leptin and a decrease in insulin secretion. These hormonal changes can disrupt metabolic cycles and lead to weight gain and fat accumulation in the abdominal area [86]. Insufficient sleep and irregular CRs can lead to increased IR in obese people. A study showed that irregular sleep and waking up at inappropriate hours increased blood glucose and IR in women with polycystic ovary syndrome (PCOS) [87].

These studies reaffirm the significant role of insulin in influencing sleep quality and body metabolism by regulating the CR and the secretion of metabolic hormones. The research findings not only provide a comprehensive understanding of how disturbances in the CR, particularly in obese individuals, can lead to IR and increase the risk of diabetes and obesity but also have important implications for the prevention and management of these conditions. This reiteration of key points helps to reinforce the audience’s understanding and confidence in the research.

Hyperglycemia, and subsequently compensatory hyperinsulinemia, can lead to a release of autonomic counterregulatory hormones, such as cortisol, adrenaline, growth hormone, and glucagon, which are involved in insomnia [66, 88]. In addition, it demonstrated that high-GI diets stimulate inflammatory immune responses, alter insulin sensitivity, and cause changes in the intestinal microbiome, perhaps through colonic epithelium barrier disturbance, which may influence sleep quality [29, 88, 89]. On the other hand, high-GI food, via insulin secretion, alters the ratio of tryptophan compared to other neutral amino acids [90]. Insulin elevates the selective uptake of neutral amino acids through the muscles and causes a higher tryptophan to neutral amino acids ratio. Because tryptophan competes with neutral amino acids to enter the brain [91], this shift can lead to increased tryptophan [92]. Tryptophan is the precursor of serotonin and melatonin that contribute to sleep onset. In this way, the level of serotonin in the brain may increase after consuming carbohydrates [66, 93]. However, more studies are required to understand the association between a high-carbohydrate diet and insomnia from an automatic perspective.

Results from a review of 43 articles published in 2022 showed nearly 95% of studies reported an association between evening chronotype and at least one unhealthy dietary habit. Morning type correlated with regular intake of fresh and low-processed meals. Further, within 47% of investigations, a higher association between late types and obesity was observed [94]. Adipose tissue has a significant role in inflammatory cytokines secretion, including IL-1, IL-6, and TNF-α, which can result in low-degree chronic inflammation [7]. The cytokines categorized as “sleep-regulatory agents” could play a role in sleep adjustment [95, 96]. Based on empirical evidence, IL-1 and TNF-α have a circadian cycle, and the highest secretion of IL-6 and TNF-α is overnight, at around 01:00–02:00 AM, and can affect slow-wave sleep [97]. Also, they may be involved in the physiological regulation of sleep in humans and animals [98]. Indeed, it was observed that obese subjects had higher levels of IL-6 and TNF-α during the morning vs. the night, which was associated with sleep disorders and BMI [99]. However, more studies are necessary to understand the exact mechanisms of association between eating habits, obesity and chronotypes.

According to the findings of this report, the following recommendations can be made to improve the quality of sleep in overweight and obese women: Among these recommendations are following a healthy and balanced diet such as the Mediterranean diet, losing weight through proper nutrition and regular physical activity, eating meals with a low-GI, especially in the evening, and ensuring that you get essential nutrients such as magnesium, zinc, vitamin D, and calcium, which help the quality of sleep. Also, adjusting the CR with regular daily activities and adequate sleep can help improve sleep and reduce the adverse effects of obesity. These strategies reduce obesity-related sleep disorders and improve women’s overall health.

To our knowledge, this is the first study assessing the association between DII and DIL with CR and quality of sleep among overweight and obese women. Therefore, new evidence indicated some predictors of the relationship between diet and sleep quality. However, despite the novelty, this study has some limitations. Since the sample size of our study only included women, our results did not generalize to both genders, so additional should also consider males. In this study, the FFQ questionnaire was used for the dietary assessment of participants. However, due to the subjective type of the assessment, over- or under-reporting of food intake is not unexpected. Finally, this study was cross-sectional in design, and causal effects were not discerning. Therefore, further prospective investigations are necessary to assess these relationships over a longer duration and with a large sample size.

## Conclusion

This study showed high adherence to DII and DIL may elicit CR disruption by increasing the odds of evening versus normal chronotype. Furthermore, higher adherence to DII was associated with increased odds of poor sleep quality index in women. Therefore, we advocate more prospective and interventional studies be conducted, with larger sample sizes and diverse populations.

## Data Availability

The data that support the findings of this study are available from Dr. Khadijeh Mirzaei but restrictions apply to the availability of these data, which were used under license for the current study, and so are not publicly available. Data are however available from the authors upon reasonable request and with permission of Dr. Khadijeh Mirzaei.

## Declarations

### Statement

We state that all methods are based on the relevant guidelines and regulations.

## Abbreviations

ANOVA: Analysis of variance
ANCOVA: Analysis of covariance
BF %: Body fat percentage
BFM: Body fat mass
BMI: Body mass index
BIA: Bioelectrical impedance analysis
BP: Blood pressure
CVD: Cardiovascular disease
CR: Circadian rhythms
CRP: C-reactive protein
DII: Dietary insulin index
DIL: Dietary insulin load
IR: Insulin resistance
FBG: Fasting blood glucose
FFQ: Food frequency questionnaire
FFM: Fat free mass
FMI: Fat mass index
GI: Glycemic index
GL: Glycemic load
HC: Hip circumference
HDL: High-density lipoprotein cholesterol
IPAQ: International physical activity questionnaires
IL-6: Interleukin-6
LDL: Low-density lipoprotein cholesterol
MEQ: Morning evening questionnaire
MET: Metabolic equivalent
PSQI: Pittsburgh sleep quality index
SD: Standard deviation
TC: Total cholesterol
TG: Triglyceride
WC: Waist circumference
WHR: Waist hip ratio
